# A Turn-On and Colorimetric Probe Based on Isophorone Skeleton for Detecting Nerve Agent Mimic Diethyl Chlorophosphite

**DOI:** 10.3390/molecules28073237

**Published:** 2023-04-05

**Authors:** Xue-Shuang Yu, Mao-Mei Zhu, Rui Zuo, Yu Peng, Ya-Wen Wang

**Affiliations:** School of Chemistry, School of Life Science and Engineering, Southwest Jiaotong University, Chengdu 610031, China; xsyu@my.swjtu.edu.cn (X.-S.Y.); zhumaomei@my.swjtu.edu.cn (M.-M.Z.); zuorui@my.swjtu.edu.cn (R.Z.)

**Keywords:** fluorescent probe, DCP (diethyl chlorophosphite), isophorone, paper sensor

## Abstract

A new turn-on probe (**SWJT-20**) based on isophorone fluorophore for the detection of nerve agent mimic diethyl chlorophosphite (DCP) was designed and synthesized. **SWJT-20** could rapidly respond to DCP within 2 s using UV-Vis or fluorescent spectra, accompanied by a significant change in the solution color under visible light or UV light, which could be observed by the naked eyes. The detection limit of **SWJT-20** to DCP was as low as 8.3 nM, which is lower than those of most reported fluorescent probes for DCP detection. Additionally, **SWJT-20** could quantitatively measure DCP using ratio changes in A_427_/A_645_ in absorption spectra. Furthermore, facile paper as sensors with the visualization of colorimetric/fluorometric responses based on **SWJT-20** has been fabricated. Notably, this probe could detect DCP vapor through gas diffusion experiments.

## 1. Introduction

Nerve agents belong to organophosphorus compounds, which are highly lethal and widely used as chemical warfare agents, causing serious impacts on society and countries [1,2]. For example, the Tokyo Metro Poisonous Gas incident caused 13 people to die and poisoning of 5500 people. At the same time, this incident caused public panic and had a negative effect on the society. Nerve agents can enter the body through the respiratory tract, digestive tract, and intact skin, inhibit acetylcholinesterase in the body, and result in muscarinic symptoms, manifested as muscle weakness, ataxia, unclear speech, pupil constriction, tremor, status epilepticus, arrhythmia, respiratory failure, and even death [3,4]. Most nerve agents are colorless and odorless, making them difficult to detect. Therefore, it is very urgent to develop simple and convenient methods for the detection of nerve agents, which is important for the military and for the public health system.

Nowadays, a variety of methods have been developed to detect organophosphorus nerve agents, such as enzyme inhibition-based biosensors [5,6], colorimetry [7,8], GC–MS [9,10], capillary electrophoresis [11,12], electrochemistry [13,14], surface enhanced Raman scattering [15,16], etc. However, these methods are limited by complicated sample preparation, cumbersome operation, low accuracy, expensive instruments, and inconvenient carrying. Fluorescent methods can overcome the above shortcomings, and therefore attract widespread attention from researchers [17]. In fact, the true organophosphorus nerve agents, such as sarin (GB), tabun (GA), soman (GD), and VX are odorless and tasteless, and microdose exposure to humans can cause death. Therefore, diethyl chlorophosphite (DCP) as nerve agent mimic was often used for the study rather than sarin agent due to their similar chemical structure [18,19,20,21].

At present, fluorescent probes used to detect DCP are mainly based on nucleophilic substitution reactions. In general, some nucleophiles were used to detect DCP including imino [22,23], pyridine [24,25], oxime [26,27], hydroxyl group [28,29], etc. Among them, some probes are turn-off fluorescent probes. For example, Cheng et al. developed a probe with pyridine group as the functional unit and the electron acceptor, after the addition of DCP to the solution containing probe, the transformation of the pyridine group into pyridinium salt occurred resulting in fluorescence quenching [30]. In 2018, we reported two fluorescent probes bearing a nucleophilic imine moiety for the selective detection of DCP, which demonstrated a remarkable fluorescence turn-off response and exhibited fast response times (within 10 s). The detection limit for these two fluorescent probes were 0.065 and 0.21 μM, respectively [31]. Churchill et al. reported a reversible fluorescent sensor based on fluorescein for the detection of nerve agent simulant. The reaction of nerve agent mimic with the phenol hydroxyl group of the probe forms a diphosphinate version of fluorescein, which reduces the fluorescence intensity. The fluorescence recovered after the addition of superoxide. The detection limit of the probe for DCP was 372.7 μM [32]. As turn-off fluorescence probe, the fluorescence of these probes can be disturbed by photobleach or fluorescence quencher [33,34]. Therefore, many turn-on fluorescent probes were developed to detect DCP [35,36,37,38,39,40,41,42,43,44] (Table S1, Supplementary Materials). For example, Zhao et al. synthesized a fluorescent probe, which was a novel thiourea-based rhodamine compound. The response time of the fluorescent probe to DCP was about 1200 s, and the minimum detection limit was 2.0 μM [35]. Son et al. reported a fluorescent probe based on rhodamine-deoxylactum skeleton with a low detection limit (9.66 nM), but the response time of this probe with DCP was within 20 min [37]. Our group also reported a two-site chemidosimeter based on fluorescein skeleton as fluorescent probe to detect DCP, the response time was about 100 min and the detection limit was 53.0 nM [39]. Recently, Zhang et al. reported a probe using the *o*-phenylenediamine unit as a detection site of DCP, which has a good linear relationship between the fluorescent intensity and the concentration of DCP in the range of 0−90.0 μM, and the detection limit was 0.082 μM. In addition, the response time of the fluorescent probe was within 2 min [44]. In summary, most of the reported turn-on fluorescent probes have a long response time and high detection limit. Therefore, it is still a great significance to design a turn-on fluorescent probe with fast response time and high sensitivity for the detection of DCP.

In connection with our research about DCP detection [31,39,45], herein, we designed a turn-on fluorescent probe **SWJT-20** based on the isophorone fluorophore for the detection of DCP according to our previous development to near-infrared (NIR) fluorescent probes [46,47]. The probe is able to rapidly respond to DCP within 2 s by a turn-on fluorescence mode, and has an extremely low detection limit (8.3 nM). In addition, a satisfied relationship line between the absorbances of A_427_/A_645_ and the concentration of DCP was obtained, indicating **SWJT-20** was the first probe to detect DCP by ratio changes in two peaks in absorption spectra, which can avoid the potential interference of background and improve the sensitivity and accuracy of detection. Moreover, the test strips of the probe were made for detecting DCP by color changes. Furthermore, the gas diffusion experiments were successfully used to detect DCP vapor by **SWJT-20**.

## 2. Results and Discussion

### 2.1. Design of SWJT-20

**Scheme 1:**
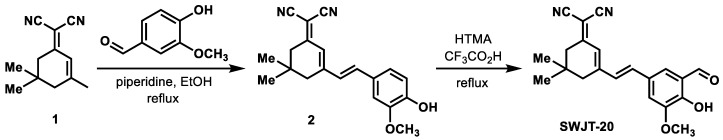
Synthesis of SWJT-20.

The hydroxyl group is a common nucleophile and is prone to react with the highly reactive chlorophosphate group of DCP [48,49]. Then, the probe **SWJT-20** was designed using isophorone as the fluorophore and the hydroxyl group as a recognition site (Scheme 1), which was synthesized by compound **2** [50]. In addition, an aldehyde group was introduced into the *ortho* position of hydroxyl group in compound **2** by Duff reaction [51] to obtain **SWJT-20** [52], which was characterized using ^1^H, ^13^C NMR spectra, and HRMS (Figures S1–S3, Supplementary Materials). The aldehyde group will form an intramolecular hydrogen bond with the *ortho* hydroxyl group, which expands the conjugation of the probe. When **SWJT-20** reacts with DCP, the hydrogen bond will be destroyed, and thus results in the changes in fluorescence.

### 2.2. Spectral Response of SWJT-20 to DCP

In order to investigate the effect of organic solvents on the fluorescence spectra of **SWJT-20**, some organic solvents were selected and examined. As shown in Figure S4 (Supplementary Materials), by comparison of fluorescence intensity at 574 nm of **SWJT-20** with DCP, the greatest change was observed in DMF. Therefore, DMF was selected as test solvent in the following experiments. Since compound **2** also has a hydroxyl group, the response of compound **2** was measured (Figure S5, Supplementary Materials). However, the changes in UV-Vis or fluorescent spectra were not apparent.

**Figure 1:**
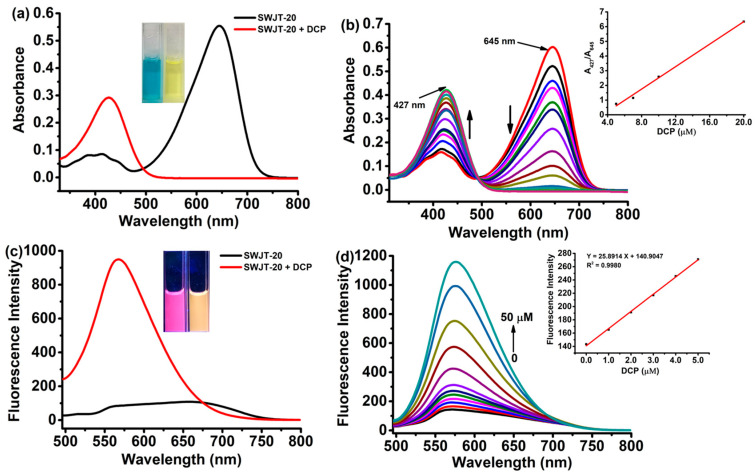
(**a**) The absorption spectra of **SWJT-20** (10.0 μM) and **SWJT-20** + DCP (50.0 μM) in DMF. Inset: Color changes under visible light before and after the addition of DCP to **SWJT-20**. (**b**) The absorption spectra of **SWJT-20** (10.0 μM) and gradual addition of DCP (0–50.0 μM) in DMF. Inset: Ratios of absorbance at 427 and 645 nm as a function of DCP concentration (5.0–20.0 μM). (**c**) The fluorescent spectra of **SWJT-20** (10.0 μM, λ_ex_ = 427 nm) and **SWJT-20** + DCP (50.0 μM) in DMF. Inset: Color changes under UV light at 365 nm before and after the addition of DCP to **SWJT-20**. (**d**) Fluorescence titrations of **SWJT-20** (10.0 μM, λ_ex_ = 427 nm) and different concentrations of DCP (0–50.0 μM) in DMF. Inset: Linear relationship between fluorescence intensity at 574 nm and the concentration of DCP (0–5.0 μM).

As shown in Figure 1a, in the UV-Vis spectra, **SWJT-20** exhibited a maximum absorption band at 645 nm, which was attributed to a formation of intramolecular hydrogen bond between hydroxyl group and the *ortho* aldehyde on the benzene ring of the probe. After the addition of DCP, the absorption band shifted to blue and a new absorption band at 427 nm appeared, which indicated that a new compound was generated and **SWJT-20** could be used to detect DCP by UV-Vis spectra. Meanwhile, the color of the solution changed from blue to yellow under visible light (Figure 1a, inset), which can be observed by the naked eyes. Moreover, with the increase in DCP concentration, the absorption peak at 645 nm gradually decreased and the peak at 427 nm gradually increased in the UV-Vis spectra (Figure 1b). Additionally, there was a good linear relationship between the absorbances of A_427_/A_645_ and the concentration of DCP in the range of 5.0−20.0 μM (Figure 1b, inset), in which changes at 427 and 645 nm were in good agreement with these color changes, as well. These results indicated that **SWJT-20** could also quantitatively measure DCP using ratio changes in A_427_/A_645_ in absorption spectra.

In the fluorescence spectra, as shown in Figure 1c, **SWJT-20** showed an emission band centered at 663 nm (*Φ* = 0.001) under the excitation of 427 nm. After the addition of DCP to the solution containing **SWJT-20**, the fluorescence at 574 nm (*Φ* = 0.044) was enhanced significantly. The fluorescent color of the solution changed from pink to orange-yellow (Figure 1c, inset), which was in good agreement with the change in fluorescent spectra. The fluorescent titration was then carried out to investigate the sensitivity of **SWJT-20** to detect DCP. The fluorescence intensity at 574 nm gradually enhanced with the increase in the concentration of DCP (Figure 1d) in the solution. A good linear relationship between DCP concentration and fluorescence intensity was plotted (Figure 1d, inset). The detection limit of the probe to DCP was then calculated to be 8.3 nM by the corresponding formula (Figure S6, Supplementary Materials), which was lower than those of most of the reported fluorescent probes for the detection of DCP. The kinetic study was then investigated by monitoring the changes in the fluorescent peak at 574 nm (Figure S7, Supplementary Materials). The fluorescence enhancement was easily observed within 2 s, and the reaction constant (*k*_obs_) could not be calculated due to this short response time [39]. Moreover, the photostabilities of probe and probe + DCP were performed (Figure S8, Supplementary Materials), which revealed that the stability of SWJT-20 and SWJT-20 + DCP was good. These results showed that **SWJT-20** was a turn-on fluorescent probe to rapidly detect DCP with high sensitivity.

**Figure 2:**
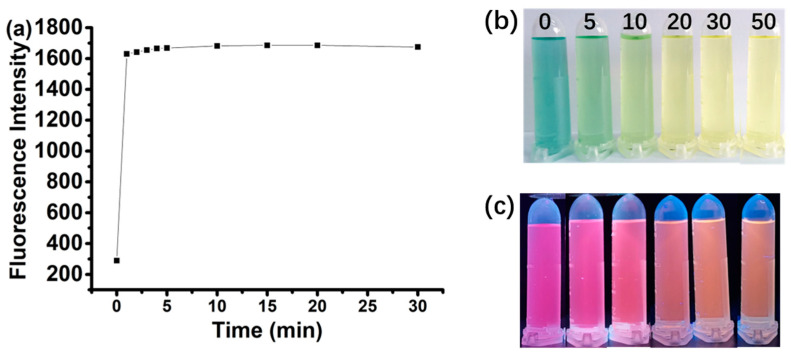
(**a**) Linear relationship between fluorescence intensity at 574 nm and response time in the concentrations of DCP (50.0 μM). (**b**) Color changes in the reaction of different concentrations of DCP (0, 5.0, 10.0, 20.0, 30.0, 50.0 μM) with **SWJT-20** under visible light. (**c**) Color changes in different concentrations of DCP (0, 5.0, 10.0, 20.0, 30.0, 50.0 μM) with **SWJT-20** under UV light.

In addition, due to the fast response time of the probe to DCP, the fluorescence stability of probe + DCP system was also measured in order to investigate whether the fluorescence of the product would weaken in a period of time. The fluorescence scanned time was 1, 2, 3, 4, 5, 10, 15, 20, and 30 min, respectively. As shown in Figure 2a, after the addition of DCP to the solution containing **SWJT-20**, the fluorescence intensity at 574 nm was almost not changed. These results indicated that the fluorescence intensity of the probe + DCP system did not decay within 30 min. Moreover, after the addition of different concentrations of DCP in the range of 0, 5.0, 10.0, 20.0, 30.0, and 50.0 μM to the solution of **SWJT-20**, the solution color changes from blue to yellow-green, and then to yellow under visible light (Figure 2b). In addition, the fluorescent color of the solution containing **SWJT-20** changed from pink to orange-red, and then to orange under UV light (Figure 2c). All of them could be observed by the naked eyes within 2 s. These results make it possible for the probe to be used for the real-time detection of DCP.

### 2.3. Selective and Competitive Experiment

In order to study the specificity of **SWJT-20** to DCP, diethyl cyanomethyl phosphate (DCMP), triethyl phosphite (TEP), nitric oxide (NO), and sodium hydrosulfide (HS^−^) were selected for the experiments (Figure S8, Supplementary Materials). In addition, acetic acid was selected to explore whether the reaction of probe with DCP was interfered under weak acidic conditions. As shown in Figure S9a (Supplementary Materials), only when DCP was added to the solution of **SWJT-20**, the fluorescence intensity of **SWJT-20** had a significant change. Furthermore, the interference study was carried out in the presence of other organophosphorus or acetic acid to the solution of **SWJT-20** with DCP (Figure S9b, Supplementary Materials). It was found that the fluorescence intensity almost did not change. These results showed that **SWJT-20** could specifically recognize DCP even in the presence of other organophosphorus disturbances or in the weak acid environment.

### 2.4. Response Mechanism

**Figure 3:**
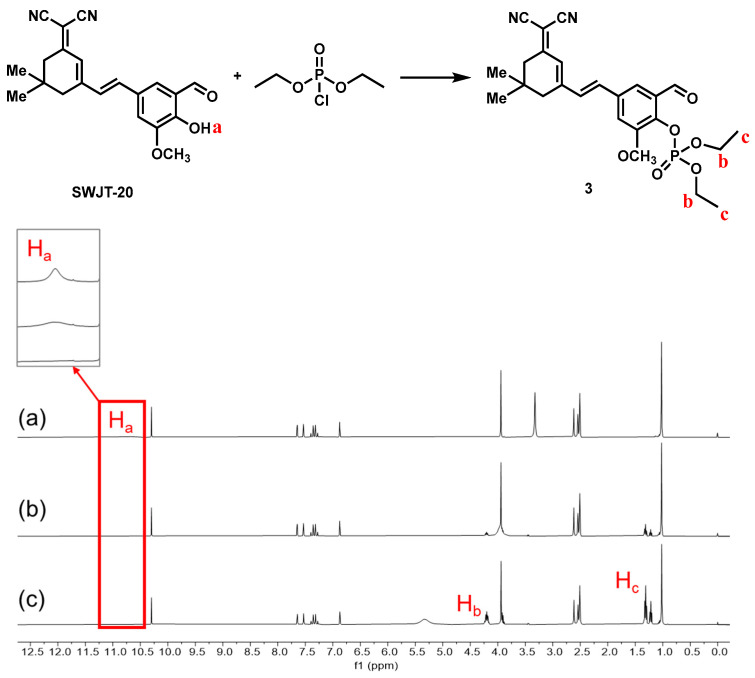
The proposed reaction mechanism and ^1^H NMR spectra of (**a**) **SWJT-20** and (**b**) **SWJT-20** + 0.5 eq. DCP (**c**) **SWJT-20** +1.0 eq. DCP in DMSO-*d*_6_.

To verify the reaction mechanism of probe **SWJT-20** with DCP, the mixture of **SWJT-20** with DCP was characterized by ^1^H and ^13^C NMR spectra (Figures S10 and S11, Supplementary Materials), which was shown to obtain compound **3** (Figure 3, top). To further investigate this mechanism, ^1^H NMR titrations were performed (Figure 3, bottom). As shown in Figure 3a, the hydrogen signal of H_a_ at 10.66 ppm belonged to the hydroxyl group of probe **SWJT-20**. After the addition of DCP to the solution of **SWJT-20**, the signal of H_a_ at 10.66 ppm decreased until it disappeared completely (Figure 3b,c). In addition, the hydrogen signal of H_b_ and H_c_ on the phosphate ester group appeared gradually at about 4.21, 1.31, and 1.22 ppm, respectively. These results also illustrated the generation of compound **3**. Meanwhile, the mixture of **SWJT-20** and DCP was characterized by LC–MS, and the peak of compound **3** at *m*/*z* 485.19 [M + H]^+^ was observed (Figure S12, Supplementary Materials). These results indicated that the electrophilic phosphate groups of DCP were attacked by the hydroxyl group, wherein a nucleophilic substitution occurred indeed between DCP and the hydroxyl group in the benzene ring of **SWJT-20**, breaking the intramolecular hydrogen bond to obtain compound **3**.

### 2.5. DFT Calculations

**Figure 4:**
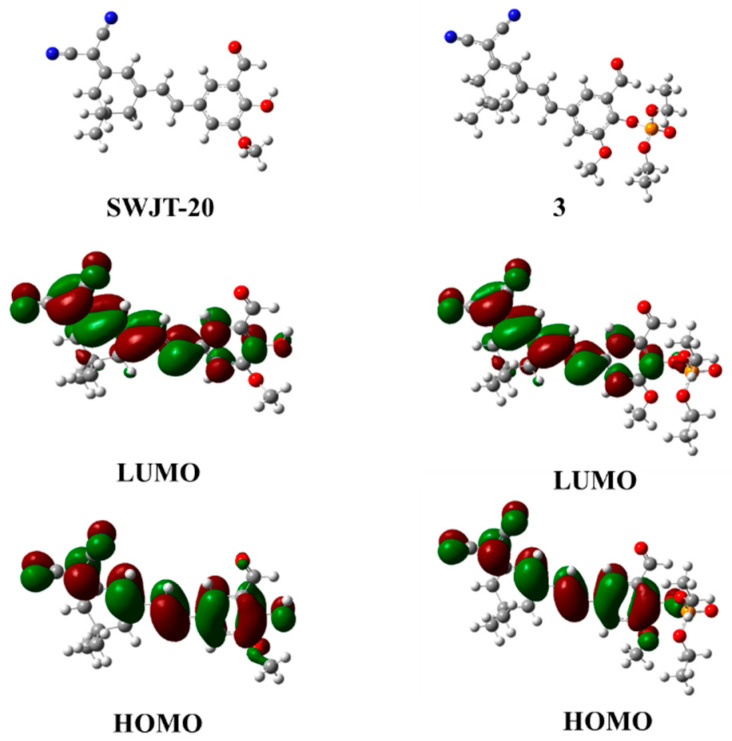
Optimal structure and frontier molecular orbital energy level plots of **SWJT-20** and compound **3**.

In order to further verify the spectral response mechanism of **SWJT-20** and DCP, density functional theory (DFT) calculations were performed using the B3LYP/6-31G method. As shown in Figure 4, the electron clouds of HOMO and LUMO were distributed equally throughout the whole large π system of **SWJT-20** including the aldehyde group and hydroxyl group, which indicated the existence of intramolecular hydrogen bond in **SWJT-20** [53,54]. As for compound **3**, the electron densities of HOMO and LUMO were similar, which were distributed to the π system except for the aldehyde group and phosphate group, indicating that there was no intramolecular hydrogen bond in compound **3**. These results suggested that the probe **SWJT-20** and compound **3** had fluorescence, which was in agreement with the fluorescence spectra.

### 2.6. Practical Applications

**Figure 5:**
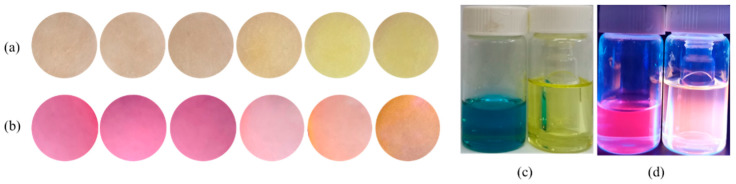
The color changes in the paper sensors at different concentrations of DCP (**a**) under visible light and (**b**) under ultraviolet light. From left to right: [DCP] = 0, 1.0 × 10^−5^ M, 1.0 × 10^−4^ M, 1.0 × 10^−3^ M, 1.0 × 10^−2^ M, 1.0 × 10^−1^ M. The color changes in **SWJT-20** (10.0 μM) dissolved in DMF and exposure to DCP vapors (**c**) under visible light and (**d**) under ultraviolet light.

Considering the practical applications, also encouraged by the results of the previous study, the probe **SWJT-20** was dissolved in DMF and loaded onto the test paper. Then, different concentrations of DCP were added. With the increase in the concentration of DCP, the color of the paper sensor changed from light brown to yellow under visible light (Figure 5a), and changed from pink to orange under a 365 nm UV lamp (Figure 5b). These results indicated that **SWJT-20** could be used as a paper sensor to detect DCP.

In addition, the ability of **SWJT-20** to recognize DCP vapor was investigated (Figure 5c,d). The solution of **SWJT-20** was placed in a closed glass vial. The color of solution was blue (Figure 5c, left) under visible light and pink (Figure 5d, left) under a 365 nm UV lamp, which could be observed by the naked eyes. In contrast, small glass bottles containing a small amount of DCP were added under the same conditions. The color of solution turned to yellow (Figure 5c, right) under visible light and light orange (Figure 5d, right) under a 365 nm UV lamp. These results showed that **SWJT-20** could detect gaseous DCP.

## 3. Material and Methods

### 3.1. Materials and Reagents

Reagents used in the experiments, such as isophorone, malonitrile, vanillin, trifluoroacetic acid, and other analytical reagents were purchased from Innochem Company, Beijing, China. All solvents, which were purchased from Tianjin Tianzheng Fine Chemical Reagent Factory (Tianjin, China), can be used directly without further purification. DMSO for fluorescence detection was spectroscopic pure.

### 3.2. Measurements

^1^H, ^13^C NMR spectra were measured by Bruker-Avb 400 spectrometer (Bruker, Bremen, Germany) using TMS as an internal parameter and DMSO-*d*_6_ as a solvent unless otherwise noted. LC–MS spectra were recorded on the Waters e2695 spectrometer (Waters, Milford, MA, USA) and chromatographic grade methanol was used as the solvent. High resolution mass spectrum (HRMS) was obtained using Bruker MicrO TOF spectrometer (Bruker, Bremen, Germany) and Bruker TI-00108 spectrometer (Bruker, Bremen, Germany). The absorption spectra were obtained using A-360 Ultraviolet spectrophotometer (AOELAB, A-360, Shanghai, China). Fluorescence spectra were obtained using a Hitachi-F7000 fluorescence spectrometer (Hitachi, F-7000, Tokyo, Japan) and the excitation and emission slit widths were 10 nm. All photographs under UV light were illuminated using a 365 nm handheld UV lamp.

### 3.3. Synthesis of Probe SWJT-20

As shown in Scheme **1**, compounds **1** and **2** were synthesized according to our previous study [50,55]. The synthetic process of **SWJT-20** was performed using a known literature approach [52].

**Synthesis of compound 1** [50,55]: Isophorone (50.02 g, 361.77 mmol) and malononitrile (23.9 g, 361.77 mmol) were placed in a 500-mL round-bottom flask and dissolved by adding 250 mL of ethanol (until no delamination). After stirring for 5 min, 5 mL of piperidine was added. The reaction mixture was stirred and refluxed for 8 h. Then, part of the solvent was removed by a rotary evaporator, and the resulting residue was crystallized overnight at 4 °C in a refrigerator. Compound **1** as a light green crystal was obtained by filtration on the next day, and the yield was 66.8%.

**Synthesis of compound 2** [50,55]: Compound **1** (9.0 g, 48.32 mmol) and 3-methoxy-4-hydroxybenzaldehyde (8.82 g, 57.98 mmol) were placed in a 250-mL round bottom flask and dissolved by adding 80 mL of ethanol. After 5 min, 2 mL of piperidine was added, and the reaction mixture was stirred and refluxed for 7 h. Then, part of the solvent was removed under vacuum, and the resulting residue was crystallized overnight at 4 °C in a refrigerator, and filtered on the next day to obtain the crude product. Thereafter, it was recrystallized with ethanol to obtain 12.8 g of red solid, which was compound **2** with a yield of 82.7%. ^1^H NMR (400 MHz, DMSO-*d*_6_): *δ* = 9.59 (s, 1H), 7.36 (s, 1H), 7.25 (d, *J* = 3.4 Hz, 2H), 7.11 (d, *J* = 8.2 Hz, 1H), 6.87–6.73 (m, 2H), 3.84 (s, 3H), 2.59 (d, *J* = 16.8 Hz, 2H), 2.54 (s, 2H), 1.02 (s, 6H) ppm.

**Synthesis of SWJT-20:** The synthetic process of **SWJT-20** was performed using a known literature [52]. Compound **2** (3.01 g, 9.36 mmol) and hexamethylenetetramine (2.5 g, 17.79 mmol) were placed in a 100-mL round bottom flask and dissolved by adding 20 mL of trifluoroacetic acid. The reaction was refluxed for 8 h. After cooling to room temperature, the mixture was extracted with CH_2_Cl_2_/H_2_O. Then, anhydrous sodium sulfate was added to dry the organic phase. After the filtered solvent was removed with a rotary evaporator, the crude product was obtained. After purification by flash column chromatography on silica gel (petroleum ether: ethyl acetate = 4:1), 0.8 g of **SWJT-20** as a red-brown solid was obtained, and the yield was 24.5%. ^1^H NMR (400 MHz, DMSO-*d*_6_): *δ* = 10.66 (s, 1H), 10.30 (s, 1H), 7.64 (d, *J* = 2.0 Hz, 1H), 7.53 (d, *J* = 1.9 Hz, 1H), 7.33 (q, *J* = 16.1 Hz, 2H), 6.87 (s, 1H), 3.94 (s, 3H), 2.61 (s, 2H), 2.54 (s, 2H), 1.02 (s, 6H) ppm. ^13^C NMR (100 MHz, DMSO-*d*_6_): *δ* = 191.2, 170.8, 156.8, 152.8, 149.4, 137.8, 128.6, 128.0, 123.0, 121.6, 115.0, 114.1, 113.7, 76.1, 56.8, 42.8, 38.7, 32.2, 27.9 (2C) ppm. HRMS (ESI): *m*/*z* calcd for C_21_H_21_N_2_O_3_ [M+H]^+^ 349.1574, found 349.1545.

### 3.4. The Preparation of the Test Stock Solution

The stock solution of **SWJT-20** (1.0 mM) was prepared in dimethyl sulfoxide. Other solutions (0.1 M), such as diethyl chlorophosphite (DCP), cyanomethyl diethyl phosphate (DCMP), and triethyl phosphite (TEP), were prepared in *N*, *N*-dimethylformamide (DMF). Acetic acid, sodium nitroprusside, and sodium hydrosulfide (0.1 M) were diluted in distilled water. Test solutions were prepared by placing 20.0 μL of the **SWJT-20** stock solution into a test tube and diluting to 2.0 mL with an organic solvent, followed by the addition of a moderate amount of other stock solutions. The mixture was then poured into a colorimetric dish and excited at 427 nm to obtain fluorescence spectra, the emission peaks in the 490–800 nm range, as well as the excitation and emission slit widths at 10/10 nm. The fluorescence quantum yield was determined with rhodamine B as the standard.

### 3.5. Fluorescence Quantum Yield Calculation

We calculated the fluorescence quantum yield of **SWJT-20** using rhodamine B as the standard and its quantum yield of 0.89 in ethanol solution [56]. The formula is as follows:

$$Y_{u}=Y_{s}\times \frac{F_{u}}{F_{s}}\times \frac{A_{s}}{A_{u}}$$

where *Y_u_* is the quantum yield of the sample to be tested; *Y_s_* is the quantum yield of rhodamine B; *F_u_* and *F_s_* are the emission band areas of the tested substance and dilute rhodamine B solution; *A_u_* and *A_s_* are the maximum absorbance values of the tested substance and dilute rhodamine B solution (the maximum absorbance values in the range of 0.01–0.05).

### 3.6. Determination and Calculation of the Lowest Detection Limit

The lowest detection limit was determined according to the previous literature [56]. The fluorescent intensity at 574 nm was a plot to **SWJT-20** with different concentrations of DCP in the range of 0.0–5.0 μM to obtain the slope. In addition, **SWJT-20** was determined 10 times with the same excitation, and the emission intensity at 574 nm was obtained to calculate the standard deviation. The specific calculation formula is provided in the Supporting information (Figure S6, Supplementary Materials).

### 3.7. Response Time Test Method for SWJT-20 to DCP

Since **SWJT-20** (10.0 μM) responded quite quickly to DCP, the color change was observed immediately after the addition of DCP (50.0 μM), the fluorescence was scanned every 0.01 min with a fluorescence spectrometer to collect emission peaks in the 490–800 nm range, and the emission peak at 574 nm was selected for a time curve.

### 3.8. The Fabrication of Paper Sensors

First, the probe **SWJT-20** (10.0 μM) was dissolved in DMF. Then, the filter paper was soaked in the solution containing **SWJT-20**. Thereafter, the filter paper was dried after 12 h to achieve the test paper sensor. After adding different concentrations of DCP, the color change was observed within 1 min, and then pictures were obtained under visible light and ultraviolet light.

## 4. Conclusions

In summary, a turn-on fluorescent probe **SWJT-20** with hydroxyl group as a recognition site was successfully designed to detect DCP. This new probe could rapidly respond to DCP within 2 s, and the detection limit was as low as 8.3 nM. In addition, **SWJT-20** was the first probe to detect DCP by ratio changes in A_427_/A_645_ in absorption spectra. The response mechanism of **SWJT-20** to DCP was confirmed by NMR, LC–MS, and density functional theory (DFT) calculation. The fluorescence intensity of compound **3** was stable within 30 min. Moreover, **SWJT-20** was a turn-on probe to detect DCP vapor or in solution through the significant color changes both in UV light and visible light. Furthermore, **SWJT-20** could serve as a paper sensor to detect DCP.

## Data Availability

Not applicable.

## Supplementary Materials

The following supporting information can be downloaded at: https://www.mdpi.com/article/10.3390/molecules28073237/s1. Table S1: Some of the reported fluorescent probes for the detection of DCP. Figures S1–S3: ^1^H, ^13^C NMR spectra, and HRMS of **SWJT-20**. Figure S4: Solvent screening of **SWJT-20**. Figure S5: Spectral response of compound **2** to DCP. Figure S6: The linear relationship of concentra-tion titration. Figure S7: The time response of **SWJT-20** to DCP. Figure S8: Photostability experiment. Figure S9: Selective and competitive experiment. Figures S10–S12: ^1^H, ^13^C NMR spectra, and LC–MS spectrum of **SWJT-20** + DCP.

## References

[1] De Cauwer H., Somville F.J.M.P., Joillet M. (2017). Neurological aspects of chemical and biological terrorism: Guidelines for neurologists. Acta Neurol. Belg..

[2] Okumura T., Hisaoka H., Yamada A., Ishimatsu S., Takasu N. (2004). The Tokyo subway sarin attack-lessons learned. Toxicol. Appl. Pharmacol..

[3] Stone R. (2018). How to defeat a nerve agent. Science.

[4] Guria U.N., Maiti K., Ali S.S., Gangopadhyay A., Samanta S.K., Roy K., Mandal D., Mahapatra A.K. (2020). An organic nanofibrous polymeric composite for ratiometric detection of diethyl chlorophosphate (DCP) in solution and vapor. ChemistrySelect.

[5] Jain M., Yadav P., Joshi B., Joshi A., Kodgire P. (2021). A novel biosensor for the detection of organophosphorus (OP)-based pesticides using organophosphorus acid anhydrolase (OPAA)-FL variant. Appl. Microbiol. Biotechnol..

[6] Zhu Y., Wang M., Cao J., Yu H., Cao Z., Jin M., Wang J., She Y. (2022). Research progress in the immobilization of key enzymes for pesticides residue detection. Biol. Bull..

[7] Li Y., Hou C., Lei J., Deng B., Huang J., Yang M. (2016). Detection of organophosphorus pesticides with colorimetry and computer image analysis. Anal. Sci..

[8] Liang M., Fan K., Pan Y., Jiang H., Wang F., Yang D., Lu D., Feng J., Zhao J.J., Yang L. (2013). Fe_3_O_4_ magnetic nanoparticle peroxidase mimetic-based colorimetric assay for the rapid detection of organophosphorus pesticide and nerve agent. Anal. Chem..

[9] Subramaniam R., Astot C., Juhlin L., Nilsson C., Ostin A. (2010). Direct derivatization and rapid GC-MS screening of nerve agent markers in aqueous samples. Anal. Chem..

[10] Lu X., Zhu X., Gao R., Tang H., Pei C., Wang H., Xiao J. (2022). Chemometrics-assisted analysis of chemical impurity profiles of tabun nerve agent using comprehensive two-dimensional gas chromatography-time-of-flight mass spectrometry. J. Chromatogr. A.

[11] Chen G., Lin Y.H., Wang J. (2006). Microchip capillary electrophoresis with electrochemical detection for monitoring environmental pollutants. Curr. Anal. Chem..

[12] Chen Q., Fung Y. (2010). Capillary electrophoresis with immobilized quantum dot fluorescence detection for rapid determination of organophosphorus pesticides in vegetables. Electrophoresis.

[13] Liu G.D., Lin Y.H. (2005). Electrochemical stripping analysis of organophosphate pesticides and nerve agents. Electrochem. Commun..

[14] Liu G., Wang J., Barry R., Petersen C., Timchalk C., Gassman P.L., Lin Y. (2008). Nanoparticle-based electrochemical immunosensor for the detection of phosphorylated acetylcholinesterase: An exposure biomarker of organophosphate pesticides and nerve agents. Chem. Eur. J..

[15] Zhao Q., Liu G., Zhang H., Zhou F., Li Y., Cai W. (2017). SERS-based ultrasensitive detection of organophosphorus nerve agents via substrate’s surface modification. J. Hazard. Mater..

[16] Wu J., Zhu Y., Gao J., Chen J., Feng J., Guo L., Xie J. (2017). A simple and sensitive surface-enhanced Raman spectroscopic discriminative detection of organophosphorous nerve agents. Anal. Bioanal. Chem..

[17] Mao T., Shi X., Lin L., Cheng Y., Luo X., Fang C. (2023). Research progress on up-conversion fluorescence probe for detection of perfluorooctanoic acid in water treatment. Polymers.

[18] Mo W., Zhu Z., Kong F., Li X., Chen Y., Liu H., Cheng Z., Ma H., Li B. (2022). Controllable synthesis of conjugated microporous polymer films for ultrasensitive detection of chemical warfare agents. Nat. Commun..

[19] Patra L., Ghosh P., Das S., Gharami S., Murmu N., Mondal T.K. (2020). A selective fluorogenic chemosensor for visual detection of chemical warfare reagent mimic diethylchlorophosphate. J. Photoch. A Photobio..

[20] Huang S., Wu Y., Zeng F., Sun L., Wu S. (2016). Handy ratiometric detection of gaseous nerve agents with AIE-fluorophore-based solid test strips. J. Mater. Chem. C..

[21] Kim H.J., Jang S., Ren W.X., Bartsch R.A., Sohn H., Kim J.S. (2011). Imine-functionalized, turn-on fluorophore for DCP. Sens. Actuators B Chem..

[22] Mahato M., Ahamed S., Tohora N., Sultana T., Ghanta S., Das S.K. (2023). A Coumarin151 derived ratiomteric and turn on chemosensor for rapid detection of sarin surrogate. Microchem. J..

[23] Gharami S., Aich K., Das S., Patra L., Mondal T.K. (2019). Facile detection of organophosphorus nerve agent mimic (DCP) through a new quinoline based ratiometric switch. New J. Chem..

[24] Radic B., Berend S., Vrdoljak A.L., Kukin D., Fuchs N., Kuca K. (2010). Antidotal efficacy of bispyridinium oximes against nerve agent. Toxicol. Lett..

[25] Sen B., Rabha M., Sheet S.K., Koner D., Saha N., Khatua S. (2021). Bis-heteroleptic Ru (II) polypyridine complex-based luminescent probes for nerve agent simulant and organophosphate pesticide. Inorg. Chem. Front..

[26] Ali S.S., Gangopadhyay A., Pramanik A.K., Guria U.N., Samanta S.K., Mahapatra A.K. (2019). Ratiometric sensing of nerve agent mimic DCP through in situ benzisoxazole formation. Dye. Pigment..

[27] Li D., Zong L., Li D., Sui S., Xiao Y., Zhuang B., Shen Y., Huang Z., Wu W. (2023). A salicylaldoximate-based AIE probe for the detection of the nerve agent simulant DCP. J. Mater. Chem. C.

[28] Ali S.S., Gangopadhyay A., Maiti K., Mondal S., Pramanik A.K., Guria U.N., Uddin M.R., Mandal S., Mandal D., Mahapatra A.K. (2017). A chromogenic and ratiometric fluorogenic probe for rapid detection of a nerve agent simulant DCP based on a hybrid hydroxynaphthalene-hemicyanine dye. Bioorg. Chem..

[29] Das M.K., Mishra T., Guria S., Das D., Sadhukhan J., Sarker S., Dutta K., Adhikary A., Chattopadhyay D., Adhikari S.S. (2022). Fluorometric detection of a chemical warfare agent mimic (DCP) using a simple hydroxybenzothiazole-diaminomaleonitrile based chemodosimeter. New J. Chem..

[30] Yao J., Fu Y., Xu W., Fan T., Gao Y., He Q., Zhu D., Cao H., Cheng J. (2016). Concise and efficient fluorescent probe via an intromolecular charge transfer for the chemical warfare agent mimic diethylchlorophosphate vapor detection. Anal. Chem..

[31] Khan M.S.J., Wang Y.W., Senge M.O., Peng Y. (2018). Sensitive fluorescence on-off probes for the fast detection of a chemical warfare agent mimic. J. Hazard. Mater..

[32] Jang Y.J., Murale D.P., Churchill D.G. (2014). Novel reversible and selective nerve agent simulant detection in conjunction with superoxide “turn-on” probing. Analyst.

[33] Zheng P., Cui Z., Liu H., Cao W., Li F., Zhang M. (2021). Ultrafast-response, highly-sensitive and recyclable colorimetric/fluorometric dual-channel chemical warfare agent probes. J. Hazard. Mater..

[34] Fu Y., Yu J., Wang K., Liu H., Yu Y., Liu A., Peng X., He Q., Cao H., Cheng J. (2018). Simple and efficient chromophoric-fluorogenic probes for diethylchlorophosphate vapor. ACS Sens..

[35] Li S., Zheng Y., Chen W., Zheng M., Zheng H., Zhang Z., Cui Y., Zhong J., Zhao C. (2019). Chromo-fluorogenic detection of soman and its simulant by thiourea-based rhodamine probe. Molecules.

[36] Huo B., Du M., Shen A., Li M., Lai Y., Bai X., Gong A., Yang Y. (2019). "Covalent-assembly"-based fluorescent probe for detection of a nerve-agent mimic (DCP) via Lossen rearrangement. Anal. Chem..

[37] Heo G., Manivannan R., Kim H., Son Y.-A. (2019). Liquid and gaseous state visual detection of chemical warfare agent mimic DCP by optical sensor. Dye. Pigment..

[38] Hu X., Zeng H., Chen T., Yuan H.-Q., Zeng L., Bao G.-M. (2020). Fast and visual detection of a chemical warfare agent mimic using a simple, effective and portable chemodosimeter. Sens. Actuators B Chem..

[39] Guo X., Liu C.X., Lu Y., Wang Y.W., Peng Y. (2022). A double-Site chemodosimeter for selective fluorescence detection of a nerve agent mimic. Molecules.

[40] Ali S.S., Gangopadhyay A., Pramanik A.K., Samanta S.K., Guria U.N., Manna S., Mahapatra A.K. (2018). Real time detection of the nerve agent simulant diethylchlorophosphate by nonfluorophoric small molecules generating a cyclization-induced fluorogenic response. Analyst.

[41] Goswami S., Das S., Aich K. (2015). Fluorescent chemodosimeter based on spirobenzopyran for organophosphorus nerve agent mimics (DCP). RSC Adv..

[42] Xu H., Zhang H., Zhao L., Peng C., Liu G., Cheng T. (2020). A naphthalimide-based fluorescent probe for the highly sensitive and selective detection of nerve agent mimic DCP in solution and vapor phase. New J. Chem..

[43] Sultana T., Mahato M., Tohora N., Ahamed S., Pramanik P., Ghanta S., Kumar Das S. (2023). A phthalimide-based turn on fluorosensor for selective and rapid detection of G-series nerve agent’s mimics. J. Photochem. Photobiol. A Chem..

[44] Chen Q., Liu J., Liu S., Zhang J., He L., Liu R., Jiang H., Han X., Zhang K. (2023). Visual and rapid detection of nerve agent mimics in gas and solution phase by a simple fluorescent probe. Anal. Chem..

[45] Feng X., Wang Y., Feng W., Peng Y. (2020). Development of BINOL-Si complexes with large stokes shifts and their application as chemodosimeters for nerve agent. Chin. Chem. Lett..

[46] Wang Y.Y., Yu X.S., Li X.J., Liu H.B., Zhu X., Wang Y.W., Peng Y. (2022). A Rapid near-infrared fluorescent probe for cysteine based on isophorone and its application in B16 Cell imaging. J. Fluoresc..

[47] Feng Y.-A., Xu H., Zhou Y., Wang B.-J., Xiao J., Wang Y.-W., Peng Y. (2022). Ratiometric detection and bioimaging of endogenous alkaline phosphatase by a NIR fluorescence probe. Sens. Actuators B Chem..

[48] Han S., Xue Z., Wang Z., Wen T.B. (2010). Visual and fluorogenic detection of a nerve agent simulant via a Lossen rearrangement of rhodamine-hydroxamate. Chem. Commun..

[49] Kim T.-I., Maity S.B., Bouffard J., Kim Y. (2016). Molecular rotors for the detection of chemical warfare agent simulants. Anal. Chem..

[50] Liu C.X., Xiao S.Y., Gong X.L., Zhu X., Wang Y.W., Peng Y. (2023). Near-infrared fluorescent probe for recognition of hypochlorite anions based on dicyanoisophorone skeleton. Molecules.

[51] Grimblat N., Sarotti A.M., Kaufman T.S., Simonetti S.O. (2016). A theoretical study of the Duff reaction: Insights into its selectivity. Org. Biomol. Chem..

[52] Xu H., Zhang C., Zhang Y.Q., Suo S.N., Wang Y.W., Peng Y.A. (2022). A red-NIR fluorescent probe for rapid and visual detection of acrolein. Chem. Commun..

[53] Zhu C., Mu A.U., Lin Y.-H., Guo Z.-H., Yuan T., Wheeler S.E., Fang L. (2016). Molecular coplanarity and self-assembly promoted by intramolecular hydrogen bonds. Org. Lett..

[54] Prochazkova E., Cechova L., Tarabek J., Janeba Z., Dracinsky M. (2016). Tunable push-pull interactions in 5-nitrosopyrimidines. J. Org. Chem..

[55] Qian M., Xia J., Zhang L., Chen Q., Guo J., Cui H., Kafuti Y.S., Wang J., Peng X. (2020). Rationally modifying the dicyanoisophorone fluorophore for sensing cysteine in living cells and mice. Sens. Actuators B Chem..

[56] Xu H., Wu S.A., Lin N.J., Xiao J., Wang Y.W., Peng Y. (2022). A NIR fluorescent probe based on C=N isomerization for fast detection of toxic acrolein. Sens. Actuators B.

