# Supplementary material for: A Turn-On and Colorimetric Probe Based on Isophorone Skeleton for Detecting Nerve Agent Mimic Diethyl Chlorophosphite
Source: Molecules. 2023 Apr 5;28(7):3237. doi: 10.3390/molecules28073237 (PMC10096706; doi:10.3390/molecules28073237)
Supplement: Supplementary file 1 [file molecules-28-03237-s001.zip › molecules-2317850-supplementary.pdf]

A Turn-On and Colorimetric Probe Based on Isophorone skeleton for Detecting Nerve Agent Mimic Diethyl Chlorophosphite

Xue-Shuang Yu, Mao-Mei Zhu, Rui Zuo, Yu Peng* and Ya-Wen Wang*

School of Chemistry & School of Life Science and Engineering
Southwest Jiaotong University, Chengdu 610031, China

pengyu@swjtu.edu.cn; ywwang@swjtu.edu.cn

Table of contents

1. Some of the reported fluorescent probes for the detection of DCP (**Table S1**).......S2

2. ^{1}H, ^{13}C NMR spectra and HRMS of SWJT-20 (**Figures S1-S3**)............................S4

3. Solvent screening of SWJT-20 (**Figure S4**)...S6

4. Spectral response of compound 2 to DCP (**Figure S5**). S7

5. The linear relationship of concentration titration (**Figure S6**). S8

6. The time response of SWJT-20 to DCP (**Figure S7**). S9

7. Photostability experiment (**Figure S8**) S10

8. Selective and competitive experiment (**Figure S9**). S11

9. ^{1}H, ^{13}C NMR spectra and LC-MS of SWJT-20 + DCP (**Figures S10-S12**). S12

1. Some of the reported fluorescent probes for the detection of DCP.

Table S1 Comparison of **SWJT-20** with other fluorescent probes for DCP

Probe		Ex/ Em (nm)	Response time	LOD	Ref.
A	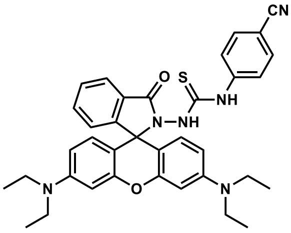	Ex= 540<br>Em= 583	within 1200 s	2×10^{-6} M	35
B	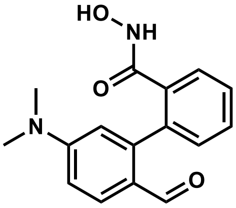	Ex= 365<br>Em= 418	within 100 s	10.4 nM	36
C	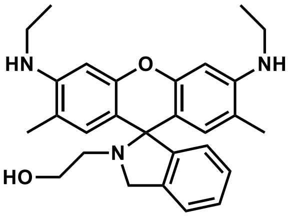	Ex= 510<br>Em= 564	20 min	9.66×10^{-9} M	37
D	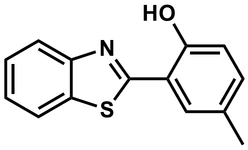	Ex= 334<br>Em= 453	within 6 s	0.186 μM	38
E	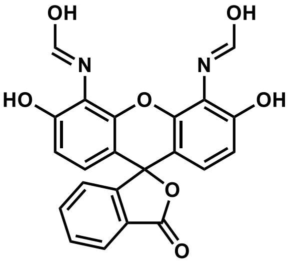	Ex= 520<br>Em= 557	within 100 min	53 nM	39
F	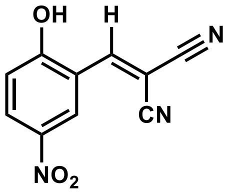	Ex= 373<br>Em= 410	within 45 s	0.1 μM	40

G	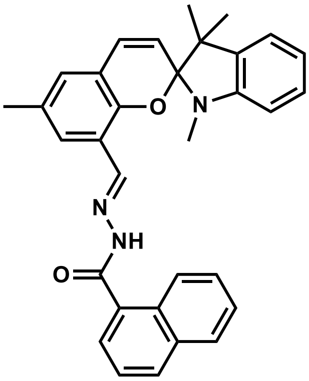	Ex=460 Em= 675	30 s	2.1×10^{-8} M	41
H	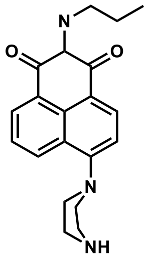	Ex=400 Em=510	5 min	5.5 nM	42
I	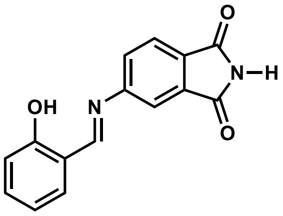	Ex=350 Em=475	150 s	1.8×10^{-7} M	43
J	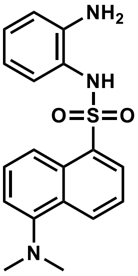	Ex= 345 Em= 515	within 2 min	0.082 μM	44
**SWJT-20**	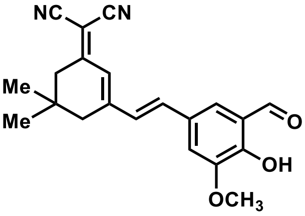	Ex= 427 Em= 574	within 2 s	8.3 nM	*This work*

2. ^1H, ^{13}C NMR spectra and HRMS of SWJT-20.

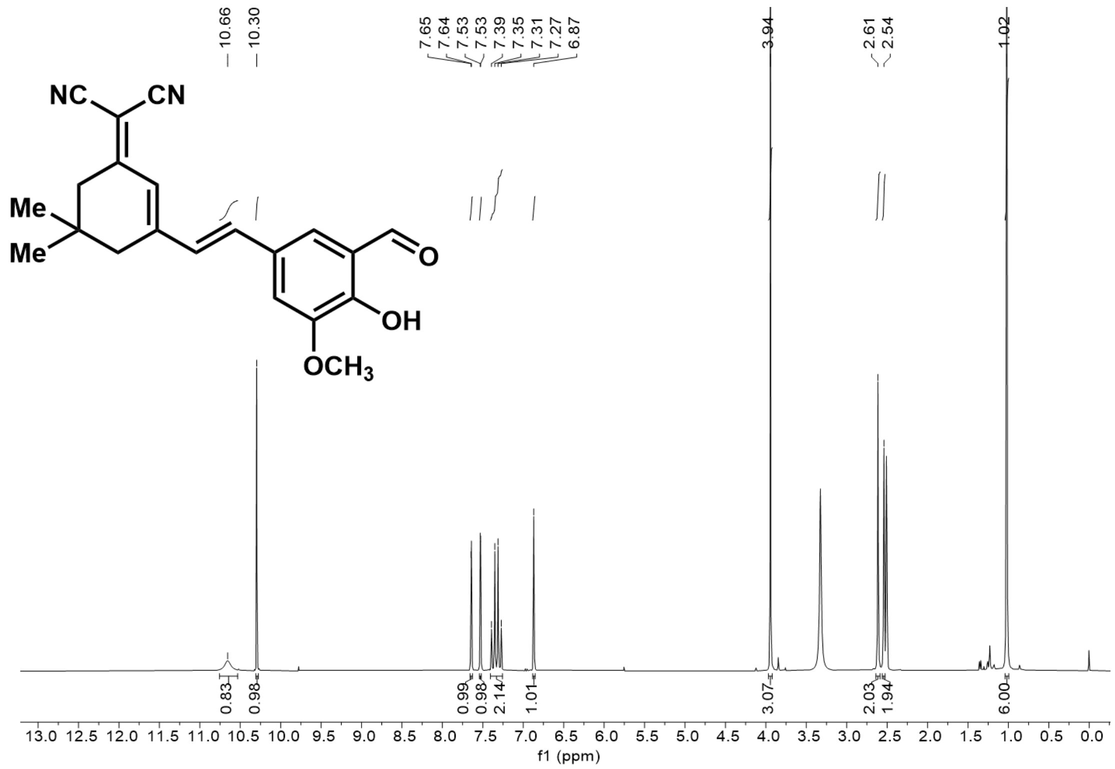

Fig. S1 ^1H NMR spectrum (400 MHz, DMSO-d_6) of **SWJT-20**.

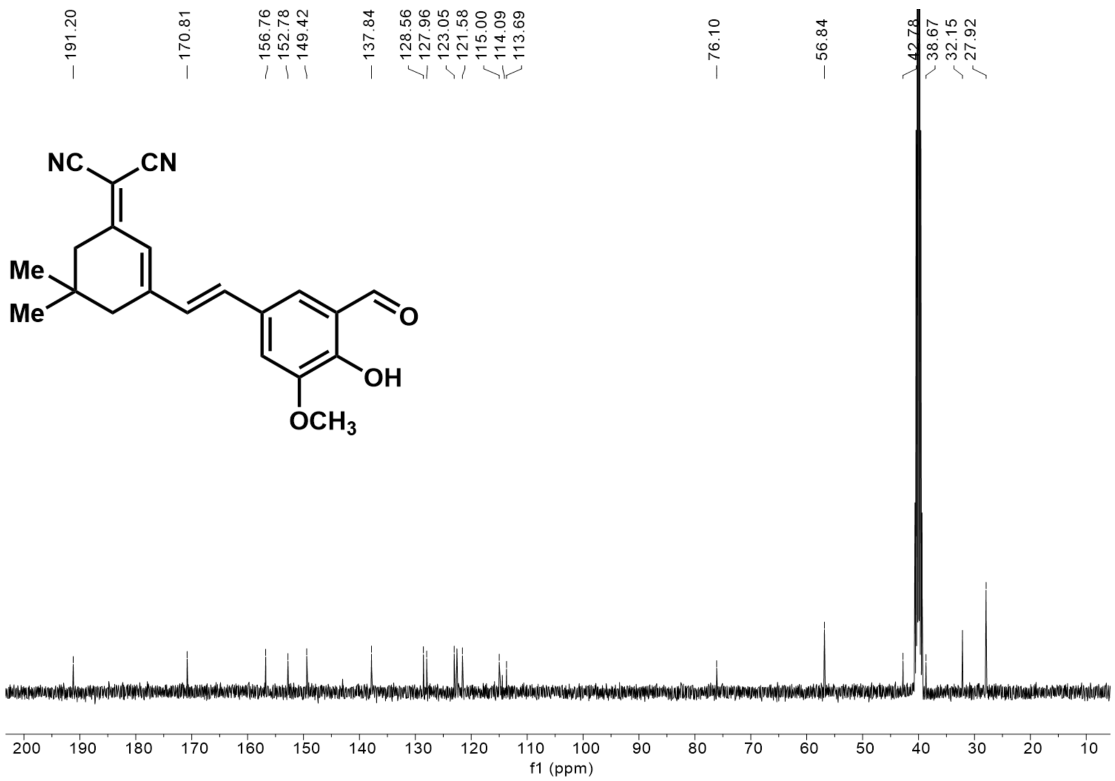

Fig. S2 ^{13}C NMR spectrum (100 MHz, DMSO-d_6) of **SWJT-20**.

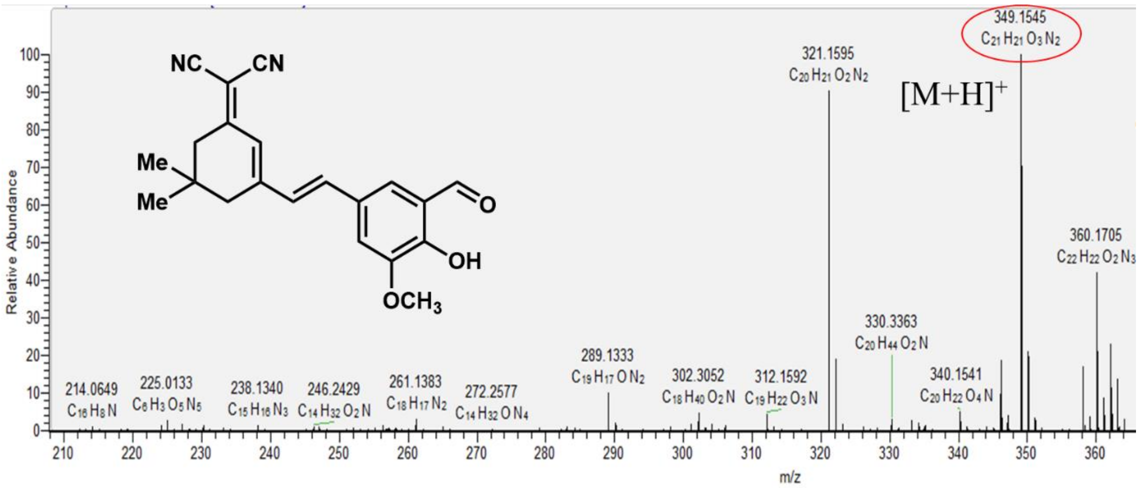

Fig. S3 HRMS of **SWJT-20**.

3. Solvent screening of SWJT-20.

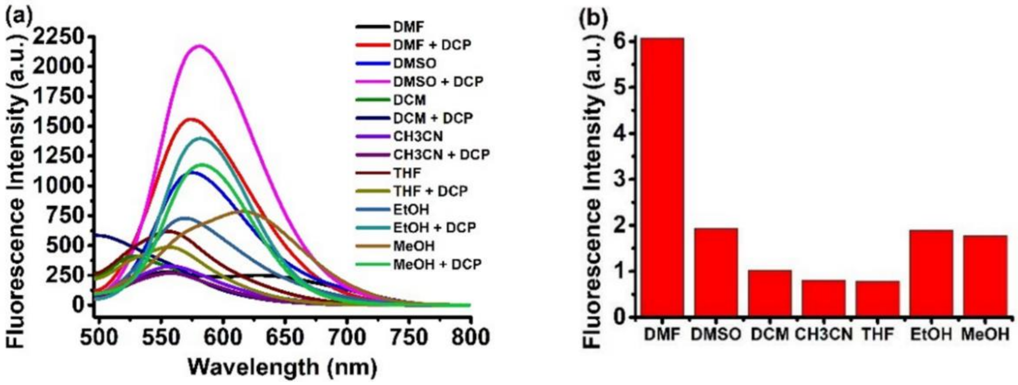

Fig. S4 (a) Fluorescence spectra of **SWJT-20** (10.0 μM, λ_{ex} = 427 nm) in various organic solvents without and with DCP; (b) Fluorescence enhancement of **SWJT-20** with and without DCP at 574 nm in different organic solvents.

4. Spectral response of compound 2 to DCP.

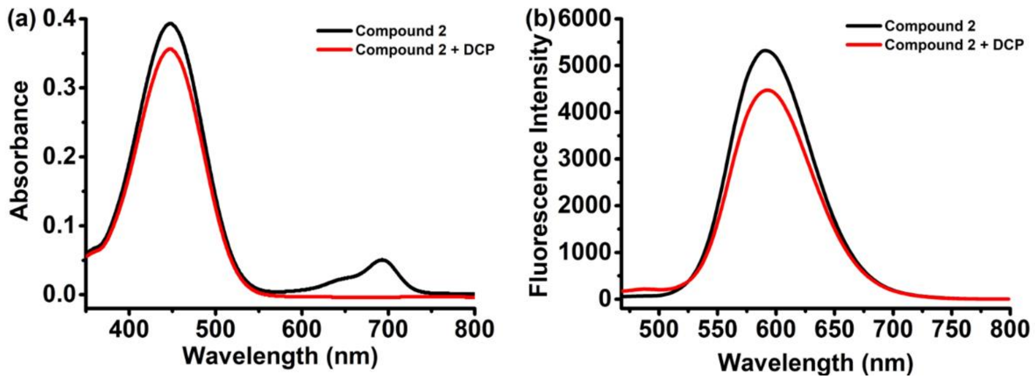

Fig. S5 Absorption(a) and fluorescence (λ_{ex} = 448 nm) (b) spectra of compound **2** (10.0 μM) with DCP (50.0 μM) in DMF.

5. The linear relationship of concentration titration.

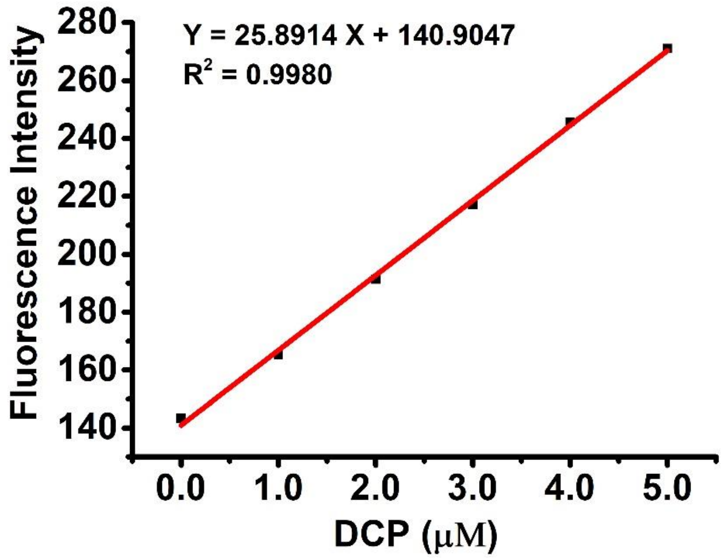

Fig. S6 Linear relationship between fluorescence intensity at 574 nm and the concentration of DCP (0-5.0 μM).

The detection limit (LOD) is calculated according to the following formula combined concentration titration linear equation.

LOD = K × δ / S   Linear Equation： Y = 25.8914 X + 140.9047

K =3; δ = 0.071(The standard deviation of the blank solution); S = 25.8914(The slope of the concentration titration curve)

LOD = 8.3 nM

6. The time response of SWJT-20 to DCP.

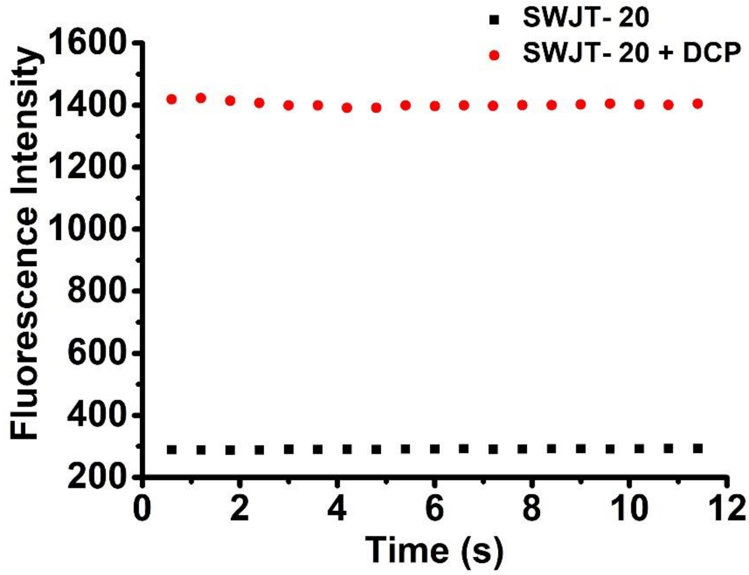

Fig. S7 Time response of fluorescence spectrum of **SWJT-20** (10.0 μM, λ_{ex} = 427 nm) and DCP (50.0 μM) in DMF at 574 nm.

7. Photostability experiment

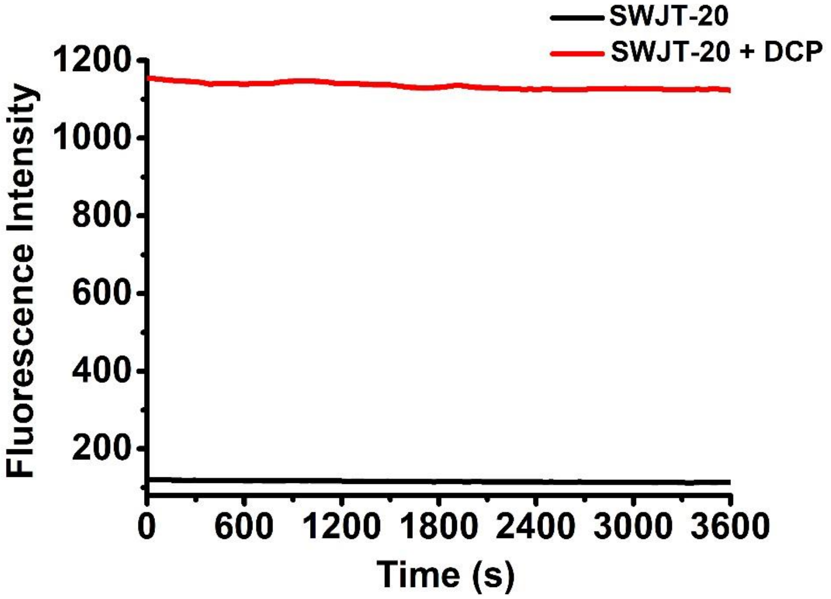

Fig. S8 Change in fluorescent intensity of SWJT-20 (10.0 μM, λ_{ex} = 427 nm) and SWJT-20 + DCP (50.0 μM) in DMF at 574 nm over an hour.

8. Selective and competitive experiment.

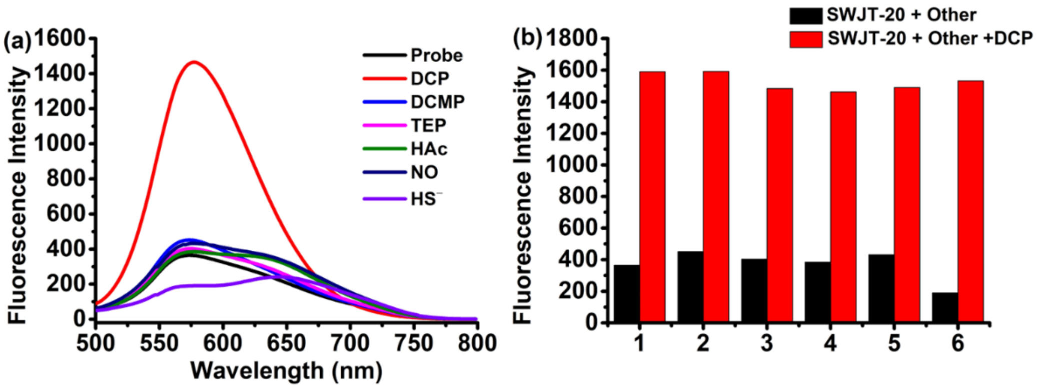

Fig. S9 The fluorescence responses of (a) **SWJT-20** (10.0 μM, λ_{ex}=427 nm) with acetic acid and other organophosphorus (50.0 μM) in DMF. (b) Fluorescence response of **SWJT-20** (10.0 μM, λ_{ex}=427 nm) at 574 nm with other substances (50.0 μM) and DCP (50.0 μM). 1. Blank, 2. DCMP, 3. TEP, 4. HAc, 5. NO, 6. HS^{-}.

9. ^{1}H, ^{13}C NMR spectra and LC-MS of SWJT-20 + DCP.

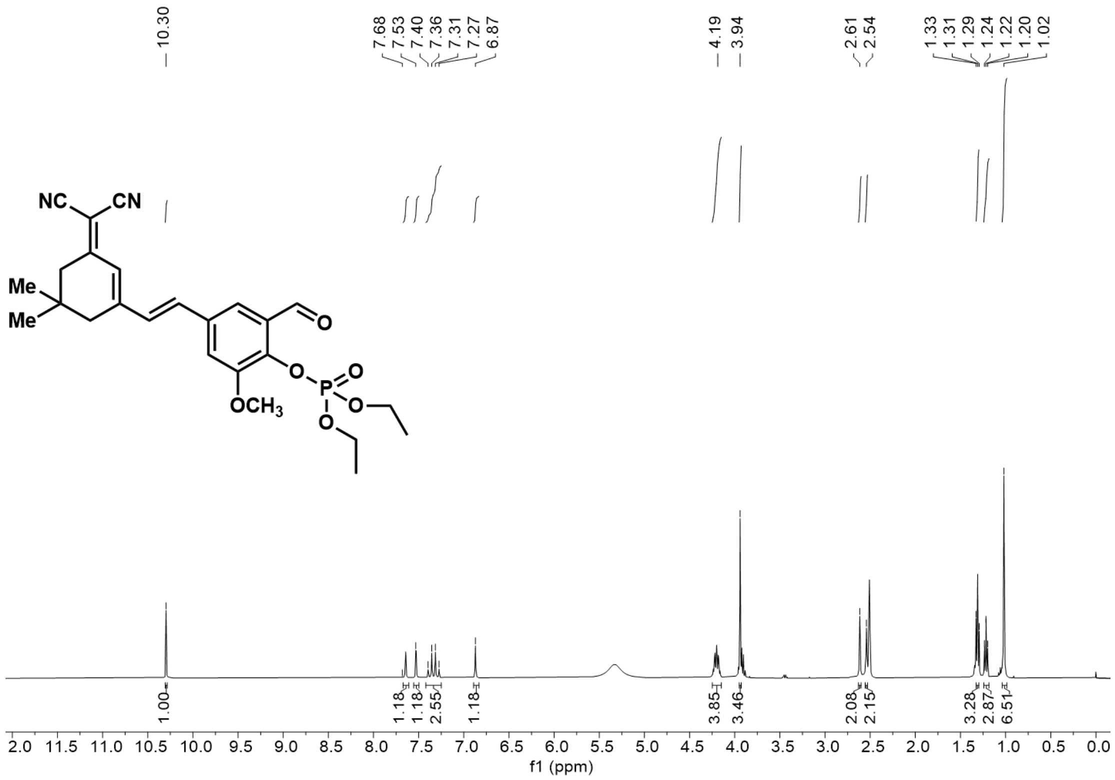

Fig. S10 ^{1}H NMR spectrum (400 MHz, DMSO-d_6) of **SWJT-20** + DCP.

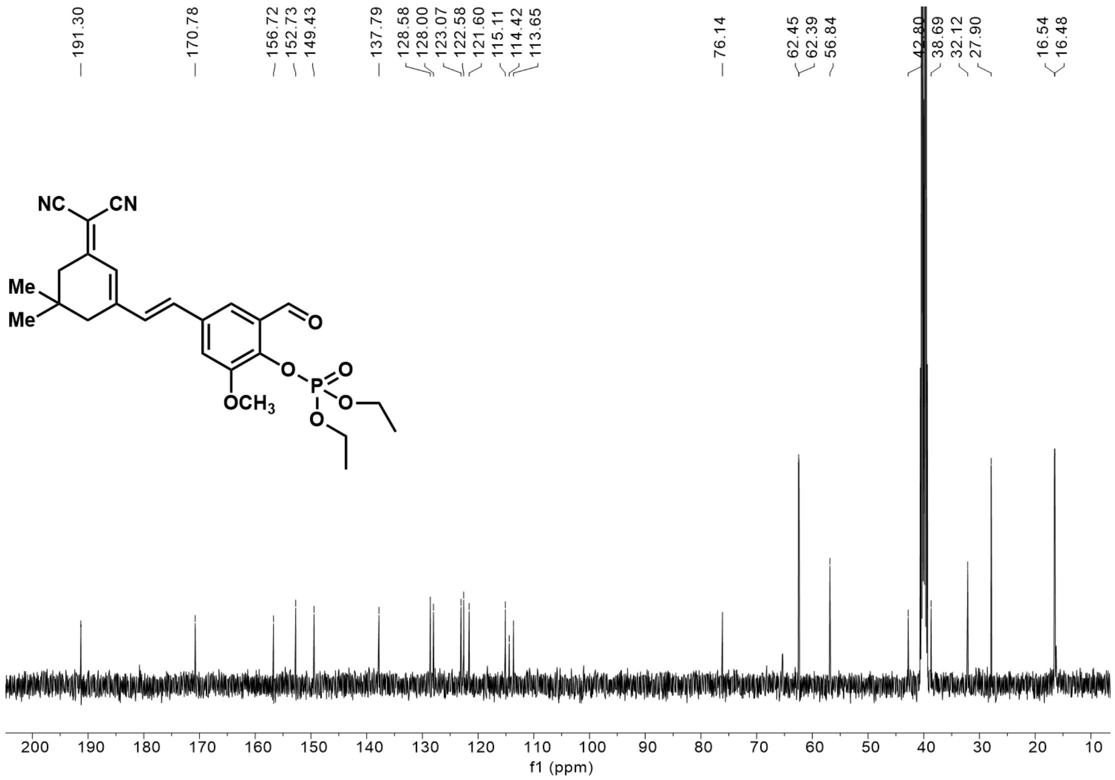

Fig. S11 ^{13}C NMR spectrum (100 MHz, DMSO-d_6) of **SWJT-20** + DCP.

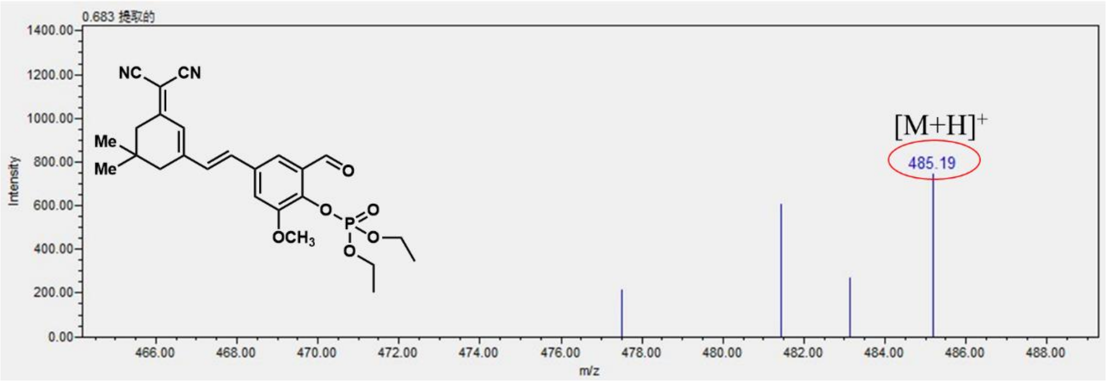

Fig. S12 LC-MS spectrum of **SWJT-20** + DCP.
